# Regulation of membrane phospholipid asymmetry by Notch-mediated flippase expression controls the number of intraepithelial TCRαβ^+^CD8αα^+^ T cells

**DOI:** 10.1371/journal.pbio.3000262

**Published:** 2019-05-09

**Authors:** Chieko Ishifune, Shin-ichi Tsukumo, Yoichi Maekawa, Katsuto Hozumi, Doo Hyun Chung, Chihiro Motozono, Sho Yamasaki, Hiroyasu Nakano, Koji Yasutomo

**Affiliations:** 1 Department of Immunology and Parasitology, Graduate School of Medicine, Tokushima University, Tokushima, Japan; 2 Department of Interdisciplinary Researches for Medicine and Photonics, Institute of Post-LED Photonics, Tokushima University, Tokushima, Japan; 3 Department of Parasitology and Infectious Diseases, Gifu University Graduate School of Medicine, Gifu, Japan; 4 Center for Highly Advanced Integration of Nano and Life Sciences (G-CHAIN), Gifu University, Gifu, Japan; 5 Department of Immunology, Tokai University School of Medicine, Isehara, Kanagawa, Japan; 6 Department of Pathology, Seoul National University College of Medicine, Seoul, Korea; 7 Department of Molecular Immunology, Research Institute for Microbial Diseases, Osaka University, Osaka, Japan; 8 Department of Molecular Immunology, Immunology Frontier Research Center, Osaka University, Osaka, Japan; 9 Department of Biochemistry, School of Medicine, Toho University, Tokyo, Japan; 10 The Research Cluster program on Immunological diseases, Tokushima University, Tokushima, Japan; Children's Hospital of Philadelphia and The University of Pennsylvania School of Medicine, UNITED STATES

## Abstract

Intestinal intraepithelial lymphocytes (IELs) expressing CD8αα on αβ T cells (TCRαβ^+^CD8αα^+^ IELs) have suppressive capabilities in enterocolitis, but the mechanism that maintains homeostasis and cell number is not fully understood. Here, we demonstrated that the number of TCRαβ^+^CD8αα^+^ IELs was severely reduced in mice lacking recombination signal binding protein for immunoglobulin kappa J region (*Rbpj*) or *Notch1* and *Notch2* in T cells. *Rbpj*-deficient TCRαβ^+^CD8αα^+^ IELs expressed low levels of *Atp8a2*, which encodes a protein with flippase activity that regulates phospholipid asymmetry of plasma membrane such as flipping phosphatidylserine in the inner leaflet of plasma membrane. *Rbpj*-deficient TCRαβ^+^CD8αα^+^ IELs cannot maintain phosphatidylserine in the inner leaflet of the plasma membrane. Furthermore, depletion of intestinal macrophages restored TCRαβ^+^CD8αα^+^ IELs in *Rbpj*-deficient mice, suggesting that exposure of phosphatidylserine on the plasma membrane in *Rbpj*-deficient TCRαβ^+^CD8αα^+^ IELs acts as an “eat-me” signal. Together, these results revealed that Notch–Atp8a2 is a fundamental regulator for IELs and highlighted that membrane phospholipid asymmetry controlled by Notch-mediated flippase expression is a critical determinant in setting or balancing the number of TCRαβ^+^CD8αα^+^ IELs.

## Abstract 2

Plasma membrane phospholipid asymmetry, mediated by the Notch-regulated flippase Atp8a2, is necessary for intestinal intraepithelial lymphocytes to escape engulfment by macrophages.

## Introduction

The intestines face various foreign antigens on their mucosal interface and thus possess an integrated immunological system that can prevent the dissemination of both commensal and pathogenic microorganisms [1][2][3]. Intraepithelial lymphocytes (IELs) are a population of lymphocytes that reside within the intestinal epithelium. IELs are classified into multiple subsets including TCRαβ^+^CD8αβ^+^, TCRαβ^+^CD8αα^+^, TCRαβ^+^CD4^+^, or T-cell receptor (TCR)γδ^+^ cells [4]. Previous studies have revealed that IELs have various roles in homeostasis of the intestine, including recovery from tissue damage and regulatory roles in suppressing colitis [5][6]. One study showed that TCRαβ^+^CD8αα^+^ IEL could prevent colitis induced by the transfer of CD4^+^CD45RB^high^ T cells to severe combined immunodeficiency (SCID) mice [5], suggesting a regulatory role for TCRαβ^+^CD8αα^+^ IELs in the mucosal immune system.

The developmental pathway of TCRαβ^+^CD8αα^+^ IEL is still poorly understood. TCRαβ^+^CD8αα^+^ IEL precursors develop by recognizing high-affinity self-antigen agonists [7][8]. Two thymic precursors of TCRαβ^+^CD8αα^+^ IEL were reported among TCRβ^+^CD4^−^CD8^−^ thymocytes, defined by dependence on transforming growth factor β (TGF-β)-activated kinase 1 (TAK1) and other markers [9]. The further differentiation of TCRαβ^+^CD8αα^+^ IELs is regulated by interleukin 15 (IL-15) or TGF-β [10] [11]. TGF-β signaling regulates the CD8α expression in thymic precursors of TCRαβ^+^CD8αα^+^ IELs [11]. The production of IL-15 from intestinal epithelial cells is required for maintaining TCRαβ^+^CD8αα^+^ IELs [12]. However, the molecular pathways that control the development or number of TCRαβ^+^CD8αα^+^ IELs remain largely undetermined.

Notch receptors interact with specific ligands that cleave the transmembrane domain of Notch [13]. The cleaved Notch translocates into the nucleus and binds to recombination signal binding protein for immunoglobulin kappa J region (Rbpj), which recruits mastermind-like 1 and p300, resulting in the up-regulation of Notch target genes [14]. Notch regulates various effector functions and development of T cells [13, 15, 16], as well as the survival of dendritic cells (DCs) and the development of macrophages [17].

In this report, we investigated whether Notch regulates the survival of intestinal immune cells. We found that the number of TCRαβ^+^CD8αα^+^ IELs is severely reduced in mice in which *Rbpj* is deleted by a CD4-*Cre* transgene. Atp8a2 is regulated by Notch signaling, and its overexpression in *Rbpj*-deficient T cells restored the number of TCRαβ^+^CD8αα^+^ IELs to levels that are comparable to those seen for wild-type mice. As Atp8a2 exhibits flippase activity that flips phosphatidylserine (PS) to the inner leaflet of the plasma membrane and thus helps keep the phospholipid asymmetry in the plasma membrane, Rbpj-deficient TCRαβ^+^CD8αα^+^ IELs had more exposed PS in their outer membrane than control cells had. Thus, Rbpj-deficient TCRαβ^+^CD8αα^+^ IELs were likely to be engulfed by intestinal macrophages. These data illustrate the existence of a novel regulatory mechanism that sets or balances the number of immune cells through flippase-mediated symmetry of membrane phospholipids.

## Results

### Rbpj in T cells is required for TCRαβ^+^CD8αα^+^ IELs

**Fig 1:**
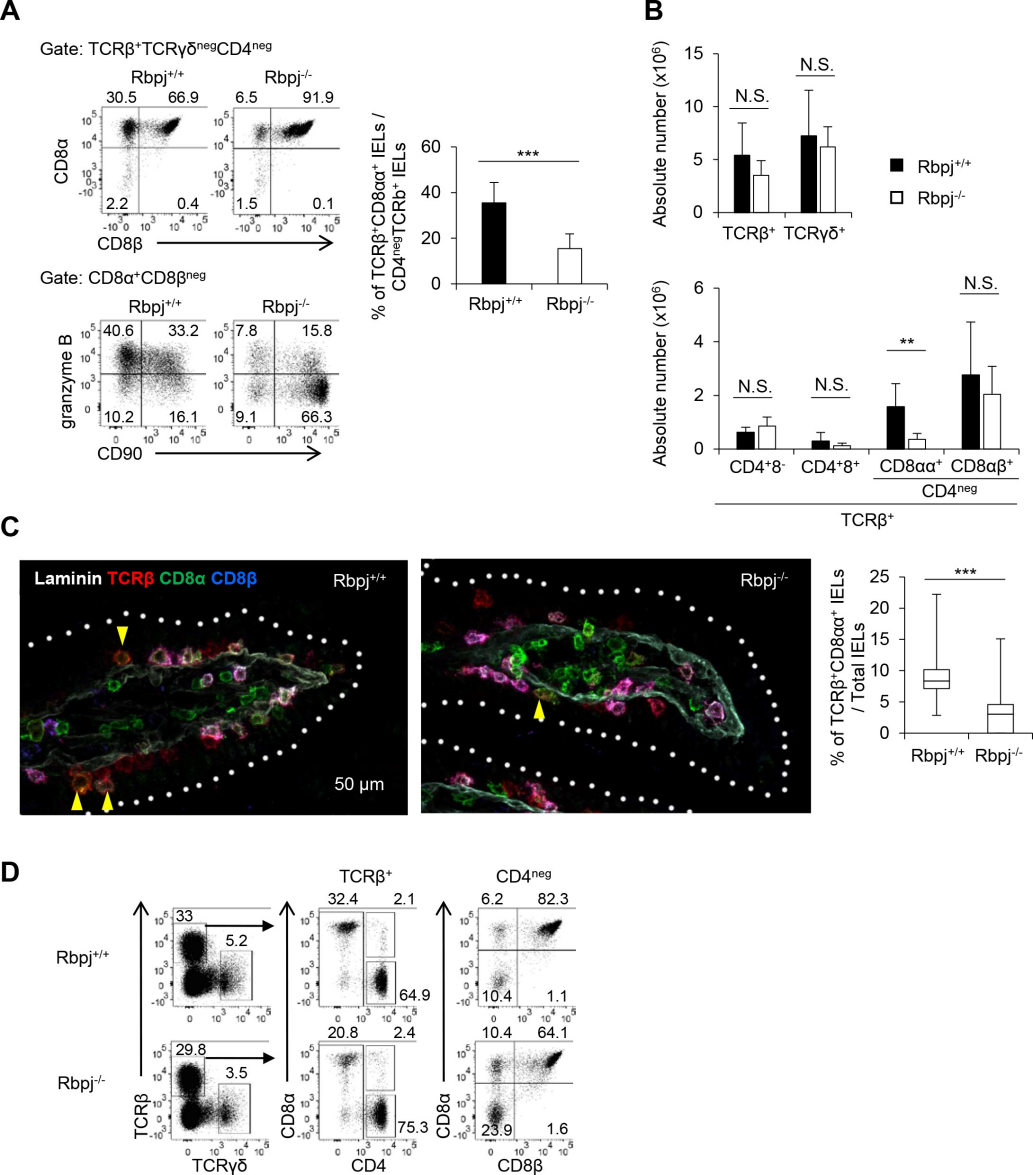
Rbpj in T cells is required for TCRαβ^+^CD8αα^+^ IELs. The (A) frequency and (B) total cell number of IELs or (A) CD90 and granzyme B expression on TCRαβ^+^CD8αα^+^ IELs in Rbpj^+/+^ and Rbpj^−/−^ mice at the age of 8 wk were evaluated by flow cytometry. The data of (A) and (B) are representative of four independent experiments. The data are shown as mean ± S.D., and ** or *** indicates *p* < 0.01 or *p* < 0.001, respectively (*n* = 5). (C) Sections of small intestine from Rbpj^+/+^ or Rbpj^−/−^ mice were stained with anti-TCRβ, CD8α, CD8β.2, and laminin, and TCRαβ^+^CD8αα^+^ IELs were detected as TCRβ^+^CD8β^-^CD8α^+^ (×400). The data are shown as mean ± S.D., and *** indicates *p* < 0.001 (*n* = 9).The data are representative of three independent experiments. (D) The number of TCRαβ^+^CD8αα^+^ cells in Rbpj^−/−^ and Rbpj^+/+^ mice in lamina propria was evaluated by flow cytometry. The data in this figure are representative of three independent experiments. Data associated with this figure can be found in the supplemental data file (S1 Data). IEL, intraepithelial lymphocyte; N.S.; not significant; Rbpj, recombination signal binding protein for immunoglobulin kappa J region; TCR, T-cell receptor.

In order to assess how Notch signaling affects the number of IELs, we analyzed IELs in the small intestine of *Rbpj*^flox/flox^ mice crossed with CD4-*Cre* transgenic mice (Rbpj^−/−^) and control *Rbpj*^+/+^ mice crossed with CD4-*Cre* transgenic (Rbpj^+/+^) mice aged 8–10 wk. TCRαβ^+^ IELs are classified into CD4^+^, CD8αβ^+^, or CD8αα^+^ cells [4] (S1 Fig). The relative and total cell number of TCRαβ^+^CD8αα^+^ IELs was about four times less in Rbpj^−/−^ than in Rbpj^+/+^ mice (Fig 1A). CD90 expression is lost during maturation of TCRαβ^+^CD8αα^+^ IELs [18]. More than 80% of TCRαβ^+^CD8αα^+^ IELs in Rbpj^−/−^ mice express CD90, compared to 50% in Rbpj^+/+^ mice. Although TCRαβ^+^CD8αα^+^ IELs acquire granzyme B expression during maturation, Rbpj^−/−^ TCRαβ^+^CD8αα^+^ IELs have a smaller number of granzyme B–positive cells. However, the total cell number of TCRαβ^+^CD4^+^ and TCRαβ^+^CD8αβ^+^ IELs was unaffected by deleting *Rbpj* (Fig 1B). The reduction in the frequency of TCRαβ^+^CD8αα^+^ IELs in Rbpj^−/−^ mice was analyzed by histological examination (Fig 1C). Sections of small intestine from Rbpj^+/+^ or Rbpj^−/−^ mice were stained with anti-TCRβ, CD8α, CD8β, and laminin, which allowed us to detect TCRαβ^+^CD8αα^+^ IELs as TCRβ^+^CD8α^+^CD8β^-^ cells. The number of TCRαβ^+^CD8αα^+^ IELs was reduced in Rbpj^−/−^ mice (Fig 1C). The number of TCRαβ^+^CD8αα^+^ T cells in the lamina propria was not decreased in Rbpj^−/−^ mice compared with control mice (Fig 1D). Although the number of TCRαβ^+^CD8αα^+^ IELs was reduced in Rbpj^−/−^ mice, the Rbpj^−/−^ and Rbpj^+/+^ mice showed comparable body weight loss after induction of colitis by dextran sodium sulfate (DSS) or 2,4,6-Trinitrobenzene sulfonic acid (TNBS) (S2 Fig).

### Intrinsic Rbpj in T cells is required for TCRαβ^+^CD8αα^+^ IELs

**Fig 2:**
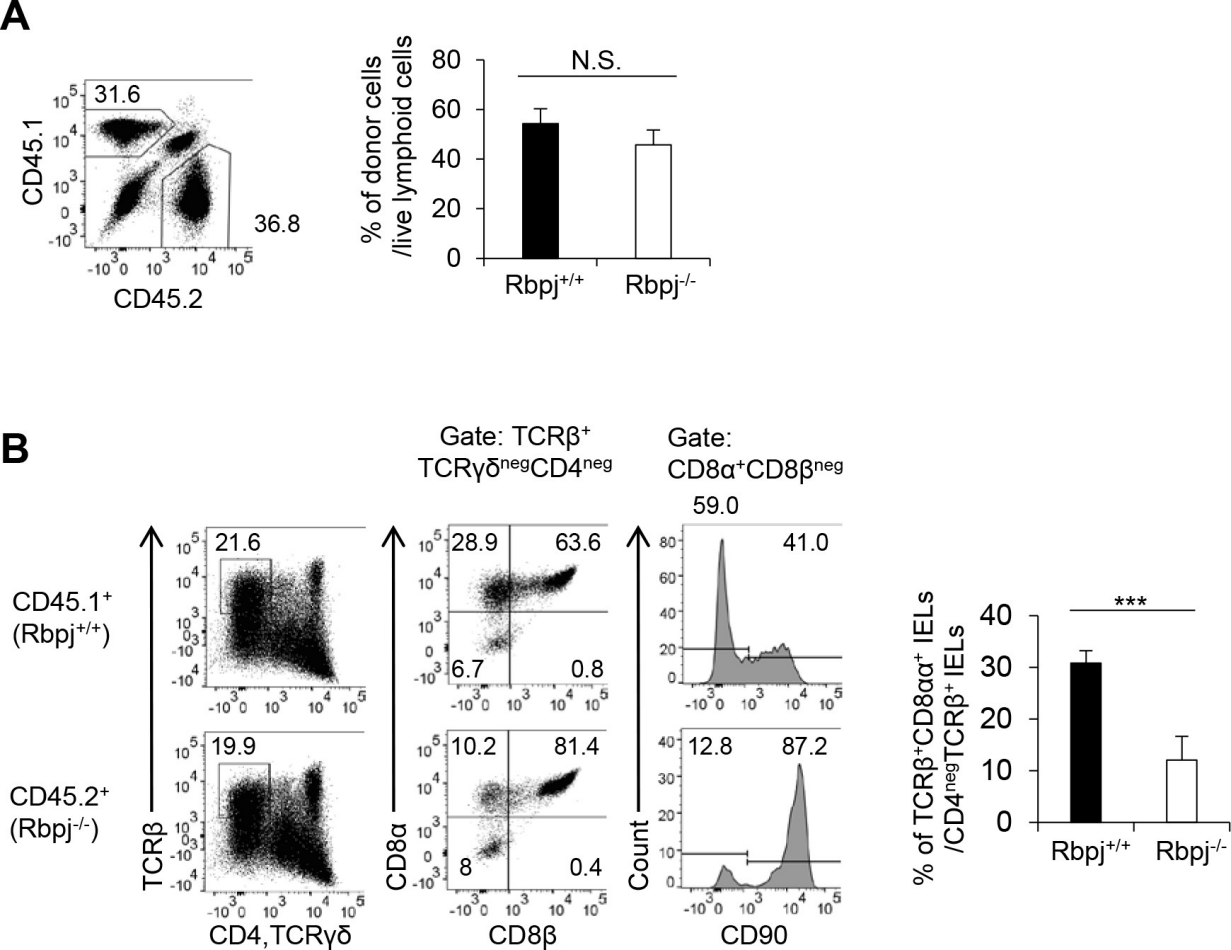
Intrinsic Rbpj in T cells is required for TCRαβ^+^CD8αα^+^ IELs. Bone marrow cells (1.5 × 10^7^) and a 1:1 ratio from Rbpj^−/−^ (CD45.2) and Rbpj^+/+^ (CD45.1) mice were transplanted into irradiated (9.5 Gy) C57BL/6 mice (CD45.1^+^CD45.2^+^). (A) The ratio of intraepithelial CD45^+^ cells between Rbpj^−/−^ and Rbpj^+/+^ mice. (B) After 6 wk posttransplantation, the number and frequency of TCRαβ^+^CD8αα^+^ IELs from Rbpj^−/−^ (CD45.2) and Rbpj^+/+^ (CD45.1) mice were evaluated. The data are shown as mean ± S.D., and *** indicates *p* < 0.001. The data in (A) and (B) are representative of three independent experiments with *n* = 5 mice in each experiment. Data associated with this figure can be found in the supplemental data file (S1 Data). IEL, intraepithelial lymphocyte; N.S.; not significant; Rbpj, recombination signal binding protein for immunoglobulin kappa J region; TCR, T-cell receptor.

To delineate whether the defect of TCRαβ^+^CD8αα^+^ IELs by *Rbpj* deficiency is cell-intrinsic, we made bone marrow chimeric mice by transferring an equal ratio of bone marrow from Rbpj^−/−^ (CD45.2) and Rbpj^+/+^ (CD45.1) mice into irradiated (CD45.1/CD45.2) mice. The ratio of total IELs was comparable between Rbpj^−/−^ and Rbpj^+/+^ mice (Fig 2A). In contrast, the relative ratio of TCRαβ^+^CD8αα^+^ IELs in Rbpj^−/−^ mice was lower than that for Rbpj^+/+^ mice (Fig 2B). Furthermore, the number of CD90^+^TCRαβ^+^CD8αα^+^ IELs was higher in Rbpj^−/−^ than Rbpj^+/+^ mice. Thus, cell-intrinsic Notch signaling is required for maintaining TCRαβ^+^CD8αα^+^ IELs.

### Notch1 and Notch2 in T cells are required for TCRαβ^+^CD8αα^+^ IELs

**Fig 3:**
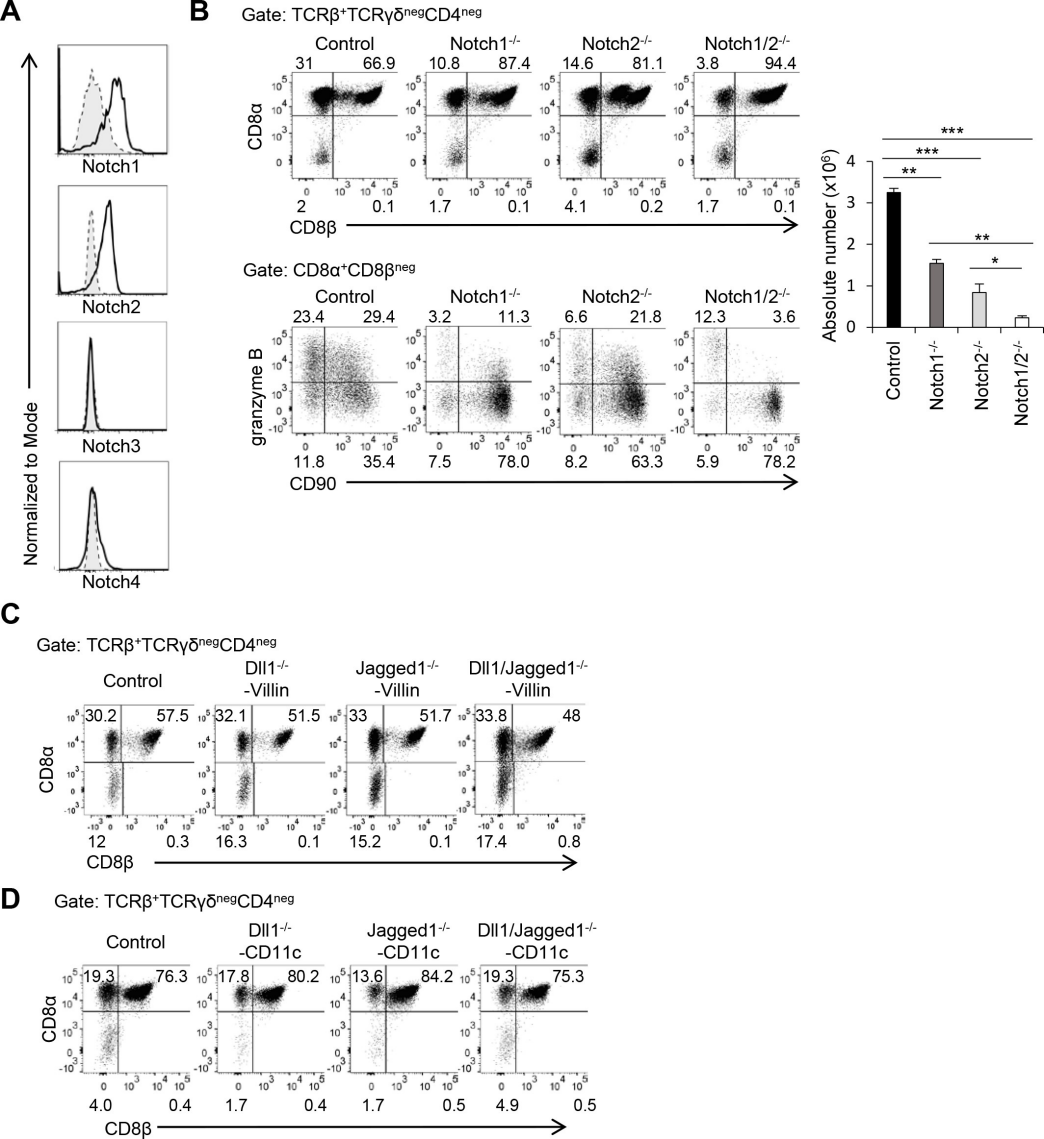
Notch1 and Notch2 are major receptors that regulate TCRαβ^+^CD8αα^+^ IELs. (A) Expression of Notch1, Notch2, Notch3, or Notch 4 on TCRαβ^+^CD8αα^+^ IELs evaluated by flow cytometry. Shadow: isotype control; solid line: Notch antibody. (B) The frequency of TCRαβ^+^CD8αα^+^ IELs or CD90/granzyme B expression on TCRαβ^+^CD8αα^+^ IELs in Notch1^−/−^, Notch2^−/−^, and Notch1/2^−/−^ mice. The data are representative of three independent experiments and are shown as mean ± S.D., and *, **, or *** indicates *p* < 0.05, *p* < 0.01, or *p* < 0.001, respectively. (C) The frequency of TCRαβ^+^CD8αα^+^ IELs in Dll1^−/−^-Villin, Jagged1^−/−^-Villin, and Dll1/Jagged1^−/−^-Villin mice. (D) The frequency of TCRαβ^+^CD8αα^+^ IELs in Dll1^−/−^-CD11c, Jagged1^−/−^-CD11c, and Dll1/Jagged1^−/−^-CD11c mice. The data in this figure are representative of three independent experiments. Data associated with this figure can be found in the supplemental data file (S1 Data). Dll1, Delta-like 1; IEL, intraepithelial lymphocyte; N.S.; not significant; Rbpj, recombination signal binding protein for immunoglobulin kappa J region.

There are four Notch receptors in mice [14], and Notch1 and Notch2—but not Notch3 and Notch4—are expressed on TCRαβ^+^CD8αα^+^ IELs (Fig 3A). We next sought to evaluate which Notch receptors control TCRαβ^+^CD8αα^+^ IELs by using *Notch1*^flox/flox^ or *Notch2*^flox/flox^ or by using *Notch1*^flox/flox^ and *Notch2*^flox/flox^ crossed with CD4-*Cre* transgenic mice (Notch1^−/−^, Notch2^−/−^, Notch1/2^−/−^, respectively). Both Notch1^−/−^ and Notch2^−/−^ mice had a reduced number of TCRαβ^+^CD8αα^+^ IELs (Fig 3B). *Notch1* and *Notch2* double deficiency further decreased the number of TCRαβ^+^CD8αα^+^ IELs compared with *Notch1* or *Notch2* single deficiency (Fig 3B). We also tested the expression of CD90 and granzyme B in TCRαβ^+^CD8αα^+^ IELs. Deficiency of either *Notch1* or *Notch2* disturbed the down-regulation of CD90 and acquisition of granzyme B expression in TCRαβ^+^CD8αα^+^ IELs (Fig 3B). Taken together, these data demonstrate that Notch1 and Notch2 cooperatively regulate the number or final differentiation of TCRαβ^+^CD8αα^+^ IELs through Rbpj.

Regarding the ligands for Notch signaling that regulate the number of TCRαβ^+^CD8αα^+^ IELs, Delta-like 1 (Dll1), Delta-like 4 (Dll4), Jagged1, and Jagged2 are expressed in intestinal epithelial cells [19]. Therefore, we deleted *Dll1*, *Jagged1*, or both genes in intestinal epithelial cells by crossing *Dll1*^flox/flox^ or *Jagged1*^flox/flox^ with Villin1-*Cre* transgenic mice (Dll1^−/−^-Villin or Jagged1^−/−^-Villin) (Fig 3C). However, the number of TCRαβ^+^CD8αα^+^ IELs of Dll1^−/−^-Villin or Jagged1^−/−^-Villin mice was not significantly different from that of control mice (Fig 3C). The double-deficient mice (Dll1/Jagged1^−/−^-Villin) also showed similar numbers of TCRαβ^+^CD8αα^+^ IELs. As DCs also express Dll1, Jagged1, and Jagged2 [20], we deleted *Dll1*, *Jagged1*, and both genes in CD11c-positive cells by crossing *Dll1*^flox/flox^ or *Jagged1*^flox/flox^ with CD11c-*Cre* transgenic mice (Dll1^−/−^-CD11c or Jagged1^−/−^-CD11c) (Fig 3D). Deficiency of *Dll1*, *Jagged1*, or both genes in DCs did not affect the number of TCRαβ^+^CD8αα^+^ IELs. These data suggest that Dll1 and Jagged1 on intestinal epithelial cells or CD11c-positive cells are not required for Notch-mediated TCRαβ^+^CD8αα^+^ IEL differentiation.

### Rbpj is not required for the development of the precursors of TCRαβ^+^CD8αα^+^ IELs

**Fig 4:**
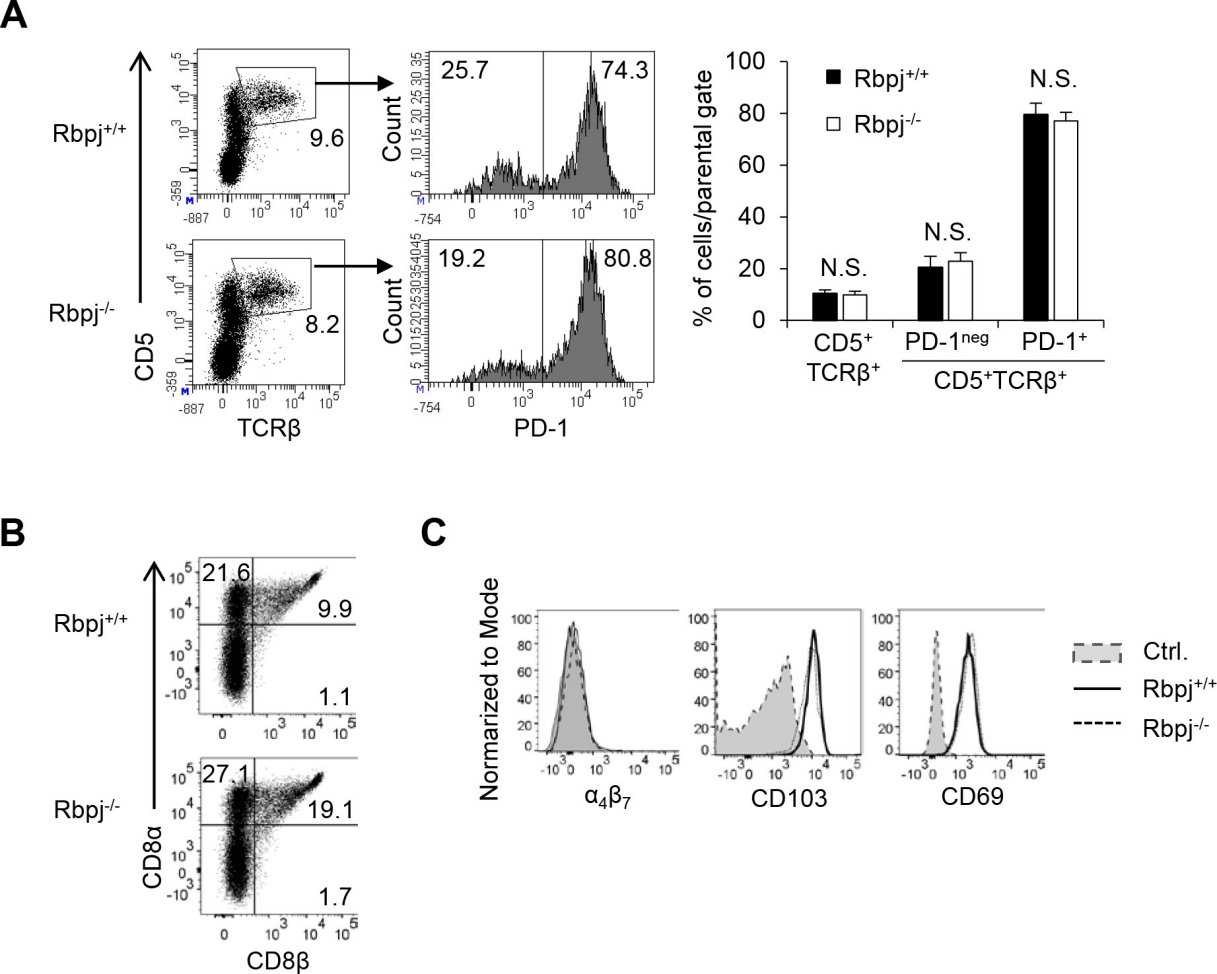
Notch deficiency does not affect the development of precursors of TCRαβ^+^CD8αα^+^ IELs. (A) The frequency and total number of PD-1^+^ and PD-1^−^ cells in CD25^−^CD1d-tetramer^−^CD4^−^CD8α^−^TCRβ^+^CD5^+^ thymocytes in Rbpj^+/+^ and Rbpj^−/−^ mice were evaluated by flow cytometry. The data are representative of three independent experiments and are shown as mean ± S.D. (B) The B220^−^NK1.1^−^CD4^−^CD8α^−^TCRγδ^−^TCRβ^+^CD5^+^ thymocytes from Rbpj^+/+^ and Rbpj^−/−^ mice were cultured in the presence of IL-15 (50 ng/ml), and the differentiation of CD8α cells was tested by flow cytometry after 8 d of culture. (C) The expression of α_4_β_7_, CD103, and CD69 on TCRαβ^+^CD8αα^+^ IELs in Rbpj^−/−^ and Rbpj^+/+^ mice. Shadow: isotype control; solid: Rbpj^+/+^; dotted: Rbpj^−/−^. The data in this figure are representative of three independent experiments. Data associated with this figure can be found in the supplemental data file (S1 Data). IEL, intraepithelial lymphocyte; N.S.; not significant; PD-1, programmed death-1; Rbpj, recombination signal binding protein for immunoglobulin kappa J region.

The TCRαβ^+^CD8αα^+^ IELs are derived from CD1d-tetramer^−^CD25^−^CD4^−^CD8α^−^TCRβ^+^CD5^+^ thymocytes [9] (S3 Fig). This precursor is further divided into programmed death-1 (PD-1)^+^ and PD-1^−^ cells [9]. We evaluated whether *Rbpj* deficiency affects the number of precursors of TCRαβ^+^CD8αα^+^ IELs. The frequency and total number of CD1d-tetramer^−^CD25^−^CD4^−^CD8α^−^TCRβ^+^CD5^+^ thymocytes were similar between Rbpj^+/+^ and Rbpj^−/−^ mice (Fig 4A). The frequency of PD-1^+^ and PD-1^−^ was also equivalent between Rbpj^+/+^ and Rbpj^−/−^ mice.

The CD4^−^CD8α^−^TCRβ^+^CD5^+^ thymocytes reexpress CD8α following stimulation with IL-15 [21]. We tested if stimulation of Rbpj-deficient NK1.1^−^B220^−^CD4^−^CD8α^−^TCRγδ^−^TCRβ^+^CD5^+^ thymocytes with IL-15 increased the reexpression of CD8α in vitro. The culture of NK1.1^−^B220^−^CD4^−^CD8α^−^TCRγδ^−^TCRβ^+^CD5^+^ thymocytes from Rbpj^+/+^ and Rbpj^−/−^ mice exhibited similarly enhanced reexpression of CD8α (Fig 4B). The expression of activation markers of IELs (CD69 [22]), a molecule required for retention of IELs within epithelium (CD103 [23]), and a molecule required for the migration of IELs to the intestine (α4β7 [24]) was similar between TCRαβ^+^CD8αα^+^ IELs from Rbpj^+/+^ and Rbpj^−/−^ mice (Fig 4C). These data suggest that Rbpj is not required for the development of precursors of TCRαβ^+^CD8αα^+^ IELs or for the further migration of TCRαβ^+^CD8αα^+^ IELs.

### Rbpj-deficient cells express low Atp8a2

**Fig 5:**
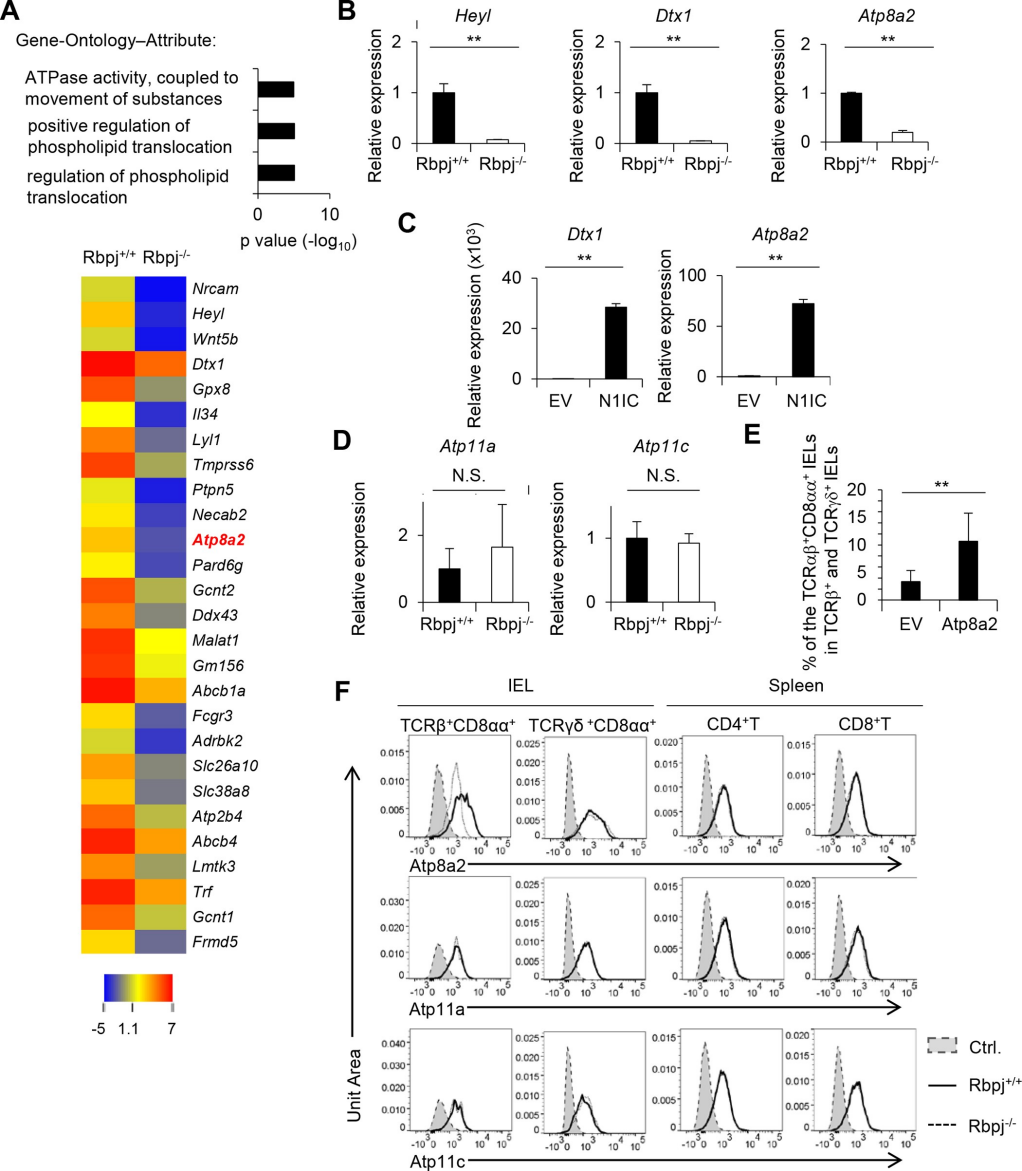
Atp8a2 is a Notch target gene in TCRαβ^+^CD8αα^+^ IELs. (A) Enriched gene ontology of genes in TCRαβ^+^CD8αα^+^ IELs. Genes for which expression is more than four times less in Rbpj^−/−^ compared with Rbpj^+/+^ mice were analyzed. A heat map of genes (>5 times reduction for *Rbpj* deficiency) in TCRαβ^+^CD8αα^+^ IELs in Rbpj^−/−^ mice compared with Rbpj^+/+^ mice is shown. (B) Expression of *Heyl*, *Dtx1*, and *Atp8a2* in TCRαβ^+^CD8αα^+^ IELs of Rbpj^+/+^ or Rbpj^−/−^ mice evaluated by real-time PCR. (C) Expression of *Dtx1* and *Atp8a2* in DO.11.10 T-cell hybridoma cells infected with control retrovirus (“EV”) or retrovirus carrying the intracellular domain of Notch1 (“N1IC”). (D) Expression of *Atp11a* and *Atp11c* in TCRαβ^+^CD8αα^+^ IELs of Rbpj^+/+^ or Rbpj^−/−^ mice evaluated by real-time PCR. (E) Bone marrow cells of Rbpj^−/−^ mice infected with control retrovirus or retrovirus carrying Atp8a2 were transplanted in irradiated wild-type mice (*n* = 4). Six weeks after transplantation, the development of TCRαβ^+^CD8αα^+^ IELs was evaluated by flow cytometry. The data in (A–E) are representative of three independent experiments and are shown as mean ± S.D., and ** indicates *p* < 0.01 (*n* = 4). (F) *Atp8a2*, *Atp11a*, and *Atp11c* expression in TCRαβ^+^CD8αα^+^ IELs, TCRγδ^+^CD8αα^+^ IELs, and splenic CD4 or CD8 T cells from Rbpj^+/+^ or Rbpj^−/−^ mice evaluated by PrimeFlow analysis. Control (“Ctrl.”); shadow; control staining, Rbpj^+/+^ mice; solid line, Rbpj^−/−^ mice; dotted line. The data in this figure are representative of three independent experiments. Data associated with this figure can be found in the supplemental data file (S1 Data). IEL, intraepithelial lymphocyte; N.S.; not significant; Rbpj, recombination signal binding protein for immunoglobulin kappa J region; TCR, T-cell receptor.

We used DNA microarray analysis to evaluate the target genes for Notch signaling that are associated with the number of TCRαβ^+^CD8αα^+^ IELs by isolating these cells from Rbpj^+/+^ and Rbpj^−/−^ mice. We performed gene ontology analysis (FuncAssociate 3.0) and found the expression of genes within three distinct categories was reduced (Fig 5A). Out of those genes, the expression of *Atp8a2* was mostly affected by deleting *Rbpj*. The reduced expression of *Atp8a2* as well as known Notch target genes *Heyl* and *Dtx1* in TCRαβ^+^CD8αα^+^ IELs from Rbpj^−/−^ mice was confirmed by real-time PCR (Fig 5B). Overexpression of the intracellular domain of Notch1 in DO.11.10 T-cell hybridoma cells markedly up-regulated the expression of *Atp8a2* together with *Dtx1* compared with control cells, suggesting the direct regulation of *Atp8a2* by Notch signaling (Fig 5C). Atp8a2 acts as a flippase [25]. Although Atp11a and Atp11c also have flippase activity at the plasma membrane [25], the expression of *Atp11a* and *Atp11c* was not reduced in *Rbpj*-deficient TCRαβ^+^CD8αα^+^ IELs compared with control cells (Fig 5D).

In order to assess whether low Atp8a2 in Rbpj^−/−^ mice is attributable to the reduced number of TCRαβ^+^CD8αα^+^ IELs, we transduced Atp8a2 using a green fluorescent protein (GFP)-expressing retrovirus into the bone marrow cells of Rbpj^−/−^ mice and transplanted the transduced cells in irradiated wild-type mice. Six weeks after transplantation, we analyzed TCRαβ^+^CD8αα^+^ IEL in bone marrow chimeric mice. The overexpression of *Atp8a2* restored the number of TCRαβ^+^CD8αα^+^ IELs by *Rbpj*-deficient bone marrow cells (Fig 5E), demonstrating that low expression of Atp8a2 by *Rbpj* deficiency is responsible for the reduced number of TCRαβ^+^CD8αα^+^ IELs in Rbpj^−/−^ mice.

Since we saw no reduction in the number of splenic CD4 and CD8 T cells in the absence of Rbpj, we next tested whether *Atp8a2* mRNA expression levels were reduced in Rbpj-deficient splenic CD4 or CD8 T cells. Depleting *Rbpj* did not affect *Atp8a2* mRNA expression in splenic CD4 and CD8 T cells or TCRγδ^+^CD8αα^+^ T cells (Fig 5F). In addition, expression levels of *Atp11a* and *Atp11c* in splenic CD4 or CD8 T cells or TCRγδ^+^CD8αα^+^ T cells were similar to those for TCRαβ^+^CD8αα^+^ IELs and were not affected by the absence of Rbpj (Fig 5F). The number of natural killer T (NKT) cells and mucosal-associated invariant T (MAIT) cells also was not affected by the absence of Rbpj (S4 Fig), which suggests that Atp8a2 has a specific role in controlling the number of TCRαβ^+^CD8αα^+^ IELs.

### Low flippase activity in Rbpj-deficient TCRαβ^+^CD8αα^+^ IELs enhances macrophage engulfment

**Fig 6:**
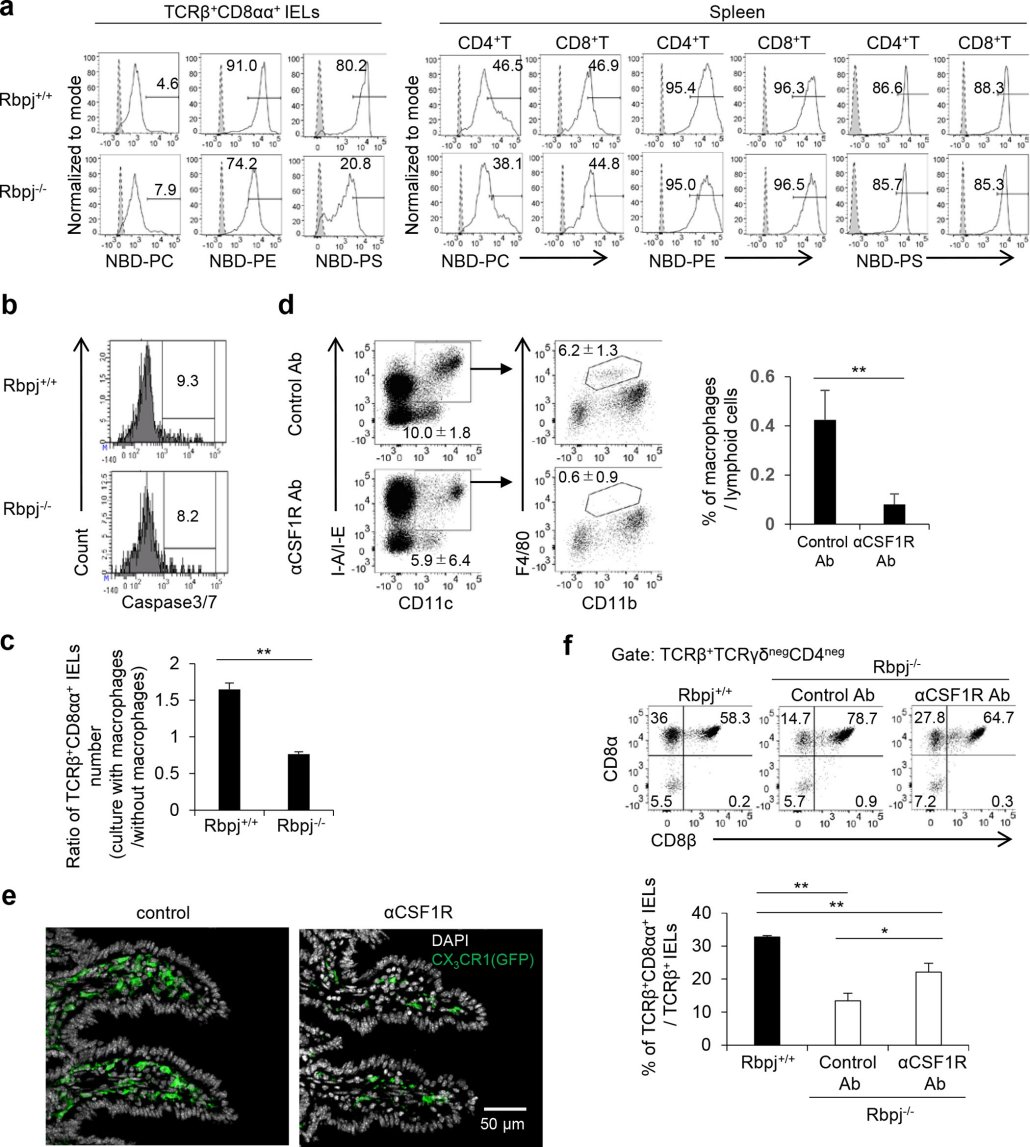
*Rbpj*-deficient TCRαβ^+^CD8αα^+^ IELs have low flippase activity. (A) TCRαβ^+^CD8αα^+^ IELs or splenic CD4^+^ or CD8α^+^ T cells from Rbpj^+/+^ or Rbpj^−/−^ mice were incubated with fluorescence-conjugated phospholipid (NBD-PS, NBD-PE, or NBD-PC), and the fluorescence was evaluated 10 min later. Shadow: unincubated control; solid line: NBD-PS, NBD-PE, or NBD-PC. (B) Expression of cleaved caspase3/7 in TCRαβ^+^CD8αα^+^ IELs from Rbpj^+/+^ or Rbpj^−/−^ mice. (C) Sorted TCRαβ^+^CD8αα^+^ IELs (CD45.2) from Rbpj^+/+^ or Rbpj^−/−^ mice were incubated with peritoneal macrophages (CD45.1/45.2) for 2 h. The number of CD45.2-positive TCRαβ^+^CD8αα^+^ IELs was counted in the presence or absence of macrophages by flow cytometry. Data are shown as the ratio of the number of TCRαβ^+^CD8αα^+^ IELs with macrophages to the number of TCRαβ^+^CD8αα^+^ IELs without macrophages. The data are representative of three independent experiments and are shown as mean ± S.D., and ** indicates *p* < 0.01. (D) Flow cytometry of CD11c^+^MHCclassII^+^CD11b^+^F4/80^+^ macrophages from the small intestine in wild-type mice treated with anti-CSF1R Ab every other day for a total of six times. Cells were analyzed 1 d after final treatment. The data are representative of three independent experiments and are shown as mean ± S.D., and ** indicates *p* < 0.01. (E) Histological section of the small intestine in CX_3_CR1^gfp/+^ mice treated with anti-CSF1R Ab every other day, for a total of two times, was stained with anti-GFP Ab (green) and DAPI (white) (×200). Mice were analyzed 1 d after final treatment. (F) Rbpj^−/−^ mice were treated with anti-CSF1R Ab every other day, for a total of six times. The number of TCRαβ^+^CD8αα^+^ IELs was evaluated 1 d after final anti-CSF1R Ab treatment. The data are representative of three independent experiments. Data are shown as mean ± S.D., and * or ** indicates *p* < 0.05 or *p* < 0.01, respectively. Data associated with this figure can be found in the supplemental data file (S1 Data). Ab, antibody; CSF1R, colony stimulating factor 1 receptor; GFP, green fluorescent protein; IEL, intraepithelial lymphocyte; NBD, 7-nitrobenz-2-oxa-1,3-diazol-4-yl; PC, phosphatidylcholine; PE, phosphatidylethanolamine; PS, phosphatidylserine; Rbpj, recombination signal binding protein for immunoglobulin kappa J region.

Atp8a2 functions as a flippase that translocates aminophospholipids, including PS and phosphatidylethanolamine (PE), from the extracellular to the cytoplasmic leaflet [25]. We examined the flippase activity of TCRαβ^+^CD8αα^+^ IELs from Rbpj^−/−^ and wild-type mice with a fluorescence-conjugated phospholipid (7-nitrobenz-2-oxa-1,3-diazol-4-yl [NBD]-PS, NBD-PE, or NBD-phosphatidylcholine [PC]). The *Rbpj*-deficient TCRαβ^+^CD8αα^+^ IELs showed lower incorporation of NBD-PS and NBD-PE compared with wild-type TCRαβ^+^CD8αα^+^ IELs (Fig 6A). In contrast, the incorporation of NBD-PC was comparable between Rbpj-deficient and wild-type cells (Fig 6A). The incorporation of NBD-PS and NBD-PE in membranes of splenic CD4^+^ or CD8α^+^ T cells isolated from Rbpj^−/−^ and Rbpj^+/+^ mice was equivalent (Fig 6A). The exposure of PS by annexin V staining showed increased numbers of annexin V–positive cells among TCRαβ^+^CD8αα^+^ IELs isolated from Rbpj^−/−^ mice compared to that for Rbpj^+/+^ mice. However, this difference was not seen for either TCRαβ^+^CD8αβ^+^ IELs or TCRαβ^+^CD4^+^ IELs from Rbpj^−/−^ and Rbpj^+/+^ mice (S5 Fig).

The expression of cleaved caspase3/7 was similar between TCRαβ^+^CD8αα^+^ IELs from Rbpj^−/−^ and Rbpj^+/+^ mice. Cell survival, B cell lymphoma 2 (Bcl-2), and Ki-67 expression was also equivalent between TCRαβ^+^CD8αα^+^ IELs from Rbpj^−/−^ and Rbpj^+/+^ mice (S6 Fig). Thus, these data indicate that increased exposure of PS on TCRαβ^+^CD8αα^+^ IELs due to *Rbpj* deficiency does not affect apoptosis and cell proliferation.

Exposure of PS on the extracellular leaflet is detected by macrophages, which results in engulfment of cells [26]. We cocultured sorted TCRαβ^+^CD8αα^+^ IELs from Rbpj^+/+^ or Rbpj^−/−^ mice with peritoneal macrophages and found increased engulfment of TCRαβ^+^CD8αα^+^ IELs from Rbpj^−/−^ mice compared to Rbpj^+/+^ mice (Fig 6C).

We then evaluated if the depletion of intestinal macrophages increased the number of TCRαβ^+^CD8αα^+^ IELs in Rbpj^−/−^ mice. The treatment of mice with anti–colony stimulating factor 1 receptor (CSF1R) antibody (Ab) effectively depletes intestinal macrophages (CD11c^+^MHC class II^+^CD11b^+^F4/80^+^), as determined by flow cytometry (Fig 6D) and histological study (CX_3_C chemokine receptor 1 [CX_3_CR1]^+^) (Fig 6E). The depletion of intestinal macrophages increased the number of TCRαβ^+^CD8αα^+^ IELs in Rbpj^−/−^ mice, although the frequency is a little bit lower than control mice (Fig 6F). As acidic phospholipids such as PS are known to regulate TCR phosphorylation via ionic interactions with the CD3 cytoplasmic domain [27][28][29], we evaluated whether Rbpj deficiency affects tonic TCR signaling. We found that the levels of phosphorylated extracellular signal–regulated kinase (p-ERK) in TCRαβ^+^CD8αα^+^ IELs from Rbpj^−/−^ mice were equivalent to those seen for Rbpj^+/+^ mice (S7 Fig). These data demonstrate the contribution of intestinal macrophages to balancing the number of TCRαβ^+^CD8αα^+^ IELs.

## Discussion

Our study provides clear evidence for the involvement of Notch signaling in regulating the number of TCRαβ^+^CD8αα^+^ IELs. The results from rescue experiments indicated that Notch controls the expression of Atp8a2, since overexpression of Atp8a2 could restore the number of TCRαβ^+^CD8αα^+^ IELs in Rbpj-deficient mice. The low levels of Atp8a2 reduced flippase activity in *Rbpj*-deficient TCRαβ^+^CD8αα^+^ IELs, which increased the engulfment of TCRαβ^+^CD8αα^+^ IELs by macrophages. Those data demonstrate that Notch-Atp8a2 is a fundamental suppressor that protects TCRαβ^+^CD8αα^+^ IELs from engulfment by intestinal macrophages. Furthermore, those data suggest that membrane phospholipid asymmetry promoted by Notch-mediated flippase expression is a crucial determinant for maintaining the number of TCRαβ^+^CD8αα^+^ IELs.

The lipid bilayers of cellular membranes show asymmetric lipid distributions. PS and PE are enriched in the cytoplasmic leaflet, and PC and sphingomyelin are major components in the exoplasmic leaflet. The flippases with type IV P-type ATPase activity are essential for the maintenance of phospholipid asymmetry in lipid bilayers. The loss of flippase function during apoptosis causes PS to be exposed in the exoplasmic leaflet, which provides an “eat-me” signal to the resident macrophages. Although Atp11a and Atp11c are cleaved by caspases during apoptosis, Atp8a2 is not, suggesting that Atp8a2 retains activity even in cells undergoing apoptosis. The naturally occurring *Atp8a2* mutant mice [30], wabbler-lethal mice, showed progressive ataxia with pronounced neurodegeneration in the central and peripheral nervous system and die in the early life period. Thus, it would be interesting to analyze the IELs from T cell–specific *Atp8a2*-deficient mice.

The depletion of macrophages in Rbpj^−/−^ mice led to the recovery of TCRαβ^+^CD8αα^+^ IELs, suggesting enhanced engulfment of *Rbpj*-deficient TCRαβ^+^CD8αα^+^ IELs by macrophages. Our in vitro coculture experiments supported increased engulfment by macrophages. Intestinal epithelial cells also have a role in clearing cells undergoing apoptosis. Bail1 is a phagocytic receptor, and Bail1-deficient mice showed pronounced colitis with many uncleared apoptotic corpses and inflammatory cytokines [31]. As the TCRαβ^+^CD8αα^+^ IELs are localized between intestinal epithelial cells, those cells should directly contact intestinal epithelial cells. However, our macrophage depletion experiments led to the recovery of TCRαβ^+^CD8αα^+^ IELs to the level of control mice. Therefore, intestinal epithelial cells might not be involved in the engulfment of TCRαβ^+^CD8αα^+^ IELs in Rbpj^−/−^ mice. This raises an important question about where *Rbpj*-deficient TCRαβ^+^CD8αα^+^ IELs are engulfed by macrophages. Macrophages in the small intestine are localized in the lamina propria as well as crypts. Even in the lamina propria, CD169^+^ macrophages reside close to the muscularis mucosa of lamina propria [32]. Furthermore, CX_3_CR1-positive macrophages that reside in lamina propria can access the intestinal lumen through forming transepithelial dendrites [33]. Two-photon microscopic analysis revealed that CD8α^+^ IELs move up and down the lateral intercellular junctions of epithelial cells and migrate between epithelial cells via the subepithelial space while occasionally accessing the lamina propria [34]. As we did not detect any reduction of CD8α^+^ in the lamina propria in our study, we speculate that at this stage, TCRαβ^+^CD8αα^+^ IELs were engulfed by macrophages after migration into the interepithelial area by the transepithelial dendrites of macrophages residing in the lamina propria.

Why is membrane asymmetry of T cells linked to TCRαβ^+^CD8αα^+^ number? One possibility is that Notch signaling is crucial for the differentiation or acquisition of effector functions of TCRαβ^+^CD8αα^+^ IELs, and cells that fail to receive the Notch signal are nonfunctional and thus removed. Indeed, *Rbpj* deficiency disturbs the differentiation from the CD90^+^ to CD90^−^ stage, which is accompanied by fewer granzyme B^+^ cells than control cells. Thus, a better understanding of the role of Notch in the acquisition of effector functions by TCRαβ^+^CD8αα^+^ IELs is needed. TCRαβ^+^CD8αα^+^ IELs have suppressive effects on naïve T-cell transfer–mediated enterocolitis. In our studies, *Rbpj*-deficient TCRαβ^+^CD8αα^+^ IELs express lower levels of granzyme B, suggesting the impairment of effector functions of TCRαβ^+^CD8αα^+^ IELs in the absence of Notch signaling. However, the reduction of TCRαβ^+^CD8αα^+^ IELs in Rbpj^−/−^ mice does not result in the spontaneous development of enterocolitis or other intestinal diseases, even in mice older than 7 mo. Notch in CD4 as well as CD8 T cells regulates crucial effector functions in multiple distinct cell types [13, 15, 16] [35–37], which might mask the development of enterocolitis in Rbpj^−/−^ mice.

It remains unclear from our studies which Notch ligands or which cells provide the Notch signal to TCRαβ^+^CD8αα^+^ IELs. Previous papers reported that Dll1, Dll4, Jagged1, and Jagged2 are expressed in epithelial cells in the small intestine. In this study, however, the deletion of Dll1 and Jagged1 in intestinal epithelial cells or CD11c-positive cells did not affect the number of TCRαβ^+^CD8αα^+^ IELs. Those data suggest that other Notch ligands or other cells are required for stimulating Notch in TCRαβ^+^CD8αα^+^ IELs.

A previous study reported that TCRαβ^+^CD8αα^+^ IELs have suppressive activity in naïve T cell–induced colitis [5]. We did not observe distinct clinical scores for DSS- or TNBS-induced colitis in control and Rbpj-deficient mice, even though the colon is the main target organ in these colitis models. We also did not observe any alterations in the degree of small intestine inflammation in Rbpj-deficient mice even when the mice were 30 wk old. Deficiency in Rbpj or Notch1 and Noth2 reduced the number of TCRαβ^+^CD8αα^+^ IELs, but residual CD8αα IELs were still detectable and could maintain intestinal homeostasis at least when the animals are relatively young. In future studies, changes in the small intestine of aged mice should be analyzed.

In summary, we have demonstrated the essential contribution of Notch in controlling the number of TCRαβ^+^CD8αα^+^ IELs and identified *Atp8a2* as the crucial target gene for this regulation. The flippase activity of Atp8a2 was required for TCRαβ^+^CD8αα^+^ IELs to prevent engulfment by gut macrophages. This novel type of regulation of IEL number might be active in other types of cells and suggests that flippase activity contributes to the control the immune cell populations.

## Methods

### Ethics statement

All animal studies were approved by the ethics committee for animal use and welfare in Tokushima University (H26-142).

### Mice

Eight- to 10-wk-old C57BL/6 mice (CD45.2) were purchased from Japan SLC (Hamamatsu, Japan). *Rbpj*^flox/flox^ [38], *Notch1*^flox/flox^ (Jackson Laboratory, Bar Harbor, MA, United States), *Notch2*^flox/flox^ [39], CD4-*Cre* recombinase transgenic [16], *Cx*_*3*_*cr1*^gfp/gfp^ (Jackson Laboratory), *Jagged1*^flox/flox^ [40], *Dll1*^flox/flox^ [41], CD11c-*Cre* transgenic (Jackson Laboratory), Villin-*Cre* transgenic (Jackson Laboratory), or C57BL/6 (CD45.1) mice were used. All mice were maintained under specific pathogen–free conditions in the animal facilities at Tokushima University, Japan.

### Flow cytometry

Fluorochrome-conjugated monoclonal Abs (mAbs) specific for mouse CD5 (53–7.3), CD8α (53–6.7), CD8β.2 (53–5.8), B220 (RA3-6B2), CD11b (M1/70), CD11c (N418), TCRβ (H57-598), TCRγδ (UC7-13D5), α_4_β_7_ (DATK32), CD45.1 (A20), CD45.2 (104), CD69 (H1.2F3), CD103 (2E7), PD-1 (29F.1A12), F4/80 (BM8), I-A-/I-E (M5/114.15.2), Notch1 (HMN1-12), Notch2 (HMN2-35), Notch3 (HMN3-133), Notch4 (HMN4-14), Ki-67 (16A8), and annexin V were purchased from BioLegend (San Diego, CA, USA). Fluorochrome-conjugated mAbs specific for mouse CD4 (RM4-5) and CD90.2 (53–2.1) were purchased from BD Biosciences (Franklin Lakes, NJ, USA). Fluorochrome-conjugated mAbs specific for mouse granzyme B (GM12) and Bcl-2 (10C4) were from Thermo Fisher Scientific (Waltham, MA, USA). Fluorochrome-conjugated mAbs specific for mouse NK1.1 (PK136) were from TONBO Biosciences (San Diego, CA, USA). PE-conjugated mAbs specific for phospho-p44/42 MAPK (Erk1/2) (Thr202/Tyr204), p44/42 MAPK (Erk1/2), and rabbit mAb IgG isotype control were from Cell Signaling (Danvers, MA, USA). CD1d-tetramer was obtained from the NIH tetramer core facility. Mouse MR1-K43A tetramers were produced as described previously [42], with modifications. Briefly, genes encoding the mouse MR1-K43A heavy chains and mouse β2m were inserted into separate pGMT7 expression plasmid and expressed in Rosetta DE3 *Escherichia coli* cells, and inclusion body protein was prepared using 0.5 mM IPTG and solubilized in 6 M Guanidine, 50 mM Tris-HCl pH 8.1, 100 mM NaCl, 2 mM EDTA. Mouse MR1 and β2m were refolded in a buffer containing 5 M urea, 100 mM Tris-HCl (pH 8.1), 2 mM Na-EDTA, 400 mM L-arginine, 6 mM cysteamine hydrochloride, 4 mM cystamine dihydrochloride and dialyzed in 10 mM Tris-HCl (pH 8.1) before FPLC purification by anion exchange. MR1 monomers were biotinylated prior to gel filtration with BirA biotin-protein ligase standard reaction kit according to manufacturer’s instructions (BirA500, Avidity) and assembled into tetramers with streptavidin-APC (Prozyme) at a streptavidin:MR1 monomer molar ratio of 1:4. The MR1 tetramers were loaded with a 3 nM of 5-OP-RU and Ac-6-FP for 16 hr at 4°C in the dark prior to use.

All samples were resuspended in PBS staining buffer containing 2% FBS and 0.01% NaN_3_, preincubated for 15 min at 4°C with 2.4G2 supernatant to block the Fc receptor, and then washed and stained with specific mAb for 20 min at 4°C. For live/dead cell analysis, 7AAD was purchased from BioLegend. Caspase3/7 and PI were identified by FAM-FLICA Caspase 3 & 7 Assay Kit, from ImmunoChemistry Technology (Bloomington, MN, USA) in accordance with the manufacturer’s protocol. For intracellular staining, cells were fixed with 4% paraformaldehyde (Wako, Japan) and permeabilized 0.1% saponin (SIGMA)-containing buffer. For staining of Ki-67, cells were treated with 70% ethanol in −20°C for 1 hr before staining with the anti-Ki-67 Ab.

To assess Erk and p-Erk levels, cells were treated with 4% paraformaldehyde for 15 min and 90% methanol for 30 min on ice before staining with anti-Erk and p-Erk Abs for 1 h at room temperature. To detect MAIT cells, cells were stained with agonist ligand (5-OP-RU) or antagonist (Ac-6-FP)-loaded APC-conjugated MR1-tetramer for 30 min on ice after treatment with Dasanitib (Axon Medchem) for 30 min at 37°C. The cells were then stained with anti-APC Ab and anti-TCRβ, B220, and I-A/I-E Abs [43]. To assess annexin V levels, cells were stained with annexin V and 7AAD for 15 min, diluted with annexin V binding buffer after staining with Abs against cell surface markers and washing twice with PBS. To detect *Atp8a2*, *Atp11a*, and *Atp11c* mRNA by flow cytometry, a PrimeFlow PCR array was performed according to the manufacturer’s protocol with a probe set containing Alexa Fluor 647-labeled Atp8a2 (VB1-3033283-PF), Atp11a (VB1-3033284-PF) Atp11c (VB1-3050975-PF), and Alexa Fluor 488–labeled Actb (VB4-10432-PF). Data were collected on a FACSCantoII (BD Biosciences) flow cytometer and analyzed using FACS Diva (BD Biosciences) or FlowJo (Tree Star, OR, USA) software.

### IELs isolation

Small intestine with the Peyer’s patches and fat tissue removed were cut vertically and then cut into small pieces. Intestines were washed with PBS and then incubated with EDTA-RMPI medium (25 ml of 1 mM EDTA, 5% FBS, 2 mM HEPES, 25 μM NaHCO_3_, and 40 μg/ml penicillin and streptomycin containing RPMI1640) at magnetic staler at 37°C for 20 min. After incubation, medium and intestines were separately collected. Intestines were further washed twice with 25 ml of 5% FBS, 2 mM HEPES, 25 μM NaHCO_3_, and 40 μg/ml penicillin and streptomycin containing RPMI 1640. After washing, there samples were collected and centrifuged at 1,800 rpm for 10 min. Cell pellets were resuspended with EDTA-RMPI medium, filtered through 190-μm nylon mesh, and then centrifuged at 1,800 rpm for 10 min. Percoll (GE Healthcare, England, United Kingdom) was diluted with 10x HBSS (SIGMA) to make 1x Percoll. Cells were resuspended with 16 ml of 40% Percoll containing EDTA-RMPI medium per 1 intestine and separated into four 4-ml samples in 15-ml tubes. Two milliliters of 75% Percoll containing EDTA-RMPI medium was added into the lower layer of 40% Percoll in each 15-ml tube. IELs were collected by harvesting the center layer between 40% and 70% Percoll after being centrifuged at 2,000 rpm for 20 min at 20°C with free-brake and low-accelerator condition.

### In vitro differentiation of IEL precursors

Thymocytes from Rbpj^+/+^ or Rbpj^−/−^ mice were purified by incubating in anti-CD4 and anti-CD8α Ab followed by BioMag goat anti-Rat IgG (QIAGEN, Hilden, Germany). The resulting double-negative thymocytes were then incubated with CD8α, CD5, TCRγδ, B220, NK1.1, TCRβ, and CD4 and sorted for CD4^−^CD8α^−^TCRγδ^−^B220^−^NK1.1^−^TCRβ^+^CD5^high^ thymocytes using a FACSAriaIII (BD Biosciences). The sorted cells were cultured in the presence of 50 ng/ml recombinant mouse IL-15 (Miltenyi Biotec) for 8 d.

### DNA microarray

TCRαβ^+^CD8αα^+^ IELs (CD4^−^TCRγδ^−^CD8β^−^) from Rbpj^−/−^ and Rbpj^+/+^ mice were sorted using a FACS AriaIII (BD Biosciences). The purity was confirmed to be >90%. RNA was extracted and genomic DNA was degraded using ReliaPrep RNA Cell Miniprep Systems from Promega (Madison, WI, USA). Quality of RNA was evaluated by Agilent 2100 BioAnalyzer. Probe preparation and microarray analyses were performed on SurePrint G3 Mouse GE 8x60K Ver.2.0 (Agilent Technologies). The resulting data were normalized using GeneSpring (Agilent Technologies) software. The data were deposited in the GEO database (GSE117122). Gene ontology study was done by FuncAssociate3.0. Genes expressed >5.0-fold up or down (*p* < 0.05) between groups were considered to be differentially expressed.

### Real-time PCR

RNA from TCRαβ^+^CD8αα^+^ IELs or DO11.10 determined to be up-regulated because of overexpression of the intracellular domain of Notch1 was extracted as described above. The cDNA was obtained by ReverTra Ace qPCR RT Master Mix with gDNA Remover from TOYOBO (Osaka, Japan). Relative expression of target genes was measured using the following primers: Atp8a2, forward: 5′-ccgagaaggatggagatgaa-3′, reverse: 5′-cggtaaacacaaagccaagc-3′; Atp11a, forward: 5′-ggagagcgaagagtgtcctg-3′, reverse: 5′-gggctgtctgtccatcaag-3′; Atp11c, forward: 5′-tattgaggctgttgccagg-3′, reverse: 5′-tttggtttccatcccagtg-3′; Dtx1, forward: 5′-ctgcacccaccaccagtaag-3′, reverse: 5′-tgtacctccgaaccacatcc-3′; Heyl, forward: 5′-aggctacaacccttcccaca-3′, reverse: 5′-gctcgtatgtctggtgctga-3′.

### Histological analysis

Small intestines were fixed in 10% sucrose (SIGMA) and 1% paraformaldehyde (WAKO) containing PBS for 1 hr followed by incubation in 10%, 20%, or 30% sucrose (Nacalai Tesque, Japan) containing PBS for 8–16 hr before being embedded in O.C.T. compound from Sakura Finetek Japan (Tokyo, Japan). The 20-μm frozen sections were fixed with ice-cold acetone (Kishida Chemical, Osaka, Japan) for 10 min and blocked with 5 mg/ml BSA/PBS. Sections were stained with Armenian hamster anti-mouse TCRβ mAb, rat anti-mouse CD8β.2 mAb, and rabbit anti-mouse laminin polyclonal Ab (Abcam, Cambridge, UK) followed by Alexa Fluor 488–conjugated donkey anti-rat IgG (Thermo Fisher Scientific), biotin-conjugated goat anti-Armenian hamster IgG (Jackson ImmunoResearch, West Grove, PA, USA), and DyLight 405-conjugated mouse anti-rabbit IgG (Jackson ImmunoResearch). After washing, sections were stained with Alexa Fluor 647–conjugated anti-mouse CD8α mAb and streptavidin-Alexa Fluor 546 (Thermo Fisher Scientific). For detection of intestinal macrophages in CX_3_CR1^gfp/+^ mice, sections were stained with anti-GFP (Thermo Fisher Scientific) followed with Alexa Fluor 488–conjugated anti-Rat IgG (Thermo Fisher Scientific). Finally, sections were enclosed with fluorescent mounting medium (Agilent, Santa Clara, CA, USA). Fluorescence in stained sections was observed by confocal laser microscopy (Nikon A1R+, Tokyo, Japan).

### Measurement of flippase activity

Total IELs were isolated from small intestine and washed twice with PBS. IELs (2 × 10^6^) were suspended in buffer (2.5 mM CaCl_2_ and 1 mM MgCl_2_ containing HBSS from WAKO) and then incubated in buffer for 10 min at 37°C with 1.5 μM NBD-labeled PS (18:1–06:0 NBD-PS), PE (18:1–06:0 NBD-PE), or PC (18:1–06:0 NBD-PC) from Avanti Polar Lipid (Alabaster, AL, USA). After incubation, 5 mg/ml fatty acid–free BSA (SIGMA)-containing HBSS was added, and IELs were incubated for 5 min on ice. IELs were washed with fatty acid–free BSA containing HBSS. Flippase activity was assessed by measuring fluorescence in the IELs.

### Bone marrow transplantation

C57BL/6 mice (CD45.1/45.2) were irradiated at 9.5 Gy. A one-to-one ratio of total bone marrow cells (1.5 × 10^7^ each) from Rbpj^+/+^ (CD45.1) or Rbpj^−/−^ (CD45.2) mice were administered into irradiated C57BL/6 mice 1 d after irradiation. The chimeric mice were analyzed 6 wk after transplantation. In some experiments, lineage-negative bone marrow cells were isolated with Lineage cell depletion kits (Miltenyi Biotec, Bergisch Gladbach, Germany). The resultant cells were infected with a retrovirus carrying *Atp8a2* or a control virus three times. The bone marrow cells were administered into 9.5 Gy–irradiated C57BL/6 mice.

### Macrophage depletion

Mice were administered intraperitoneally with anti-CFS1R Ab (clone AFS98) or Rat IgG2a Isotype control (clone 2A3) from Bio X Cell (West Lebanon, NH, USA) (400 μg/mouse) every other day, and mice were analyzed 1 d after final treatment.

### In vitro macrophage engulfment assay

A total of 1.5 ml of 4 mg/ml thioglycolate medium was injected into CD45.1/CD45.2 mice. After 4–6 d, 6 ml of ice-cold PBS was injected into the peritoneal cavity, and then macrophages were collected in PBS. Peritoneal macrophages (1.5 × 10^5^) were preincubated at 37°C in U-bottom 96-well plates. After 2 hr, sorted TCRαβ^+^ CD8αα^+^ IELs from Rbpj^+/+^ or Rbpj^−/−^ (CD45.2) were added, mixed, and then centrifuged at 600*g* for 5 min. Cells were stained with CD45.1, CD45.2, CD8α, CD8β, TCRβ, TCRγδ, CD4, and 7AAD. Live TCRαβ^+^ CD8αα^+^ IELs were counted by flow cytometry.

### Statistical analysis

For all experiments, the significant differences between groups were calculated using Student *t* test for unpaired data. Differences were considered significant when *p* < 0.05.

## Supporting information

S1 Fig Gating strategy to distinguish IEL subsets. Total IELs were stained with anti-CD8α, CD8β, TCRβ, CD4, and TCRγδ antibodies. The 7AAD-negative cells were analyzed. 7AAD, 7-aminoactinomycin D; IEL, intraepithelial lymphocyte; TCR, T-cell receptor.
(TIFF)

S2 Fig Rbpj^+/+^ and Rbpj^−/−^ mice have similar body weight loss. Body weight loss of model mice with (A) DSS-induced and (B) TNBS-induced colitis was measured. Body weight is displayed as the percentage of body weight relative to the weight at treatment initiation. Square: PBS injection; open circle: Rbpj^+/+^; closed circle: Rbpj^−/−^. The data in (A) and (B) are representative of three independent experiments and are shown as mean ± S.D. Data associated with this figure can be found in the supplemental data file (S1 Data). DSS, dextran sodium sulfate; Rbpj, recombination signal binding protein for immunoglobulin kappa J region; TNBS, 2,4,6-Trinitrobenzene sulfonic acid.
(TIFF)

S3 Fig Gating strategy to detect thymic precursors of TCRαβ^+^CD8αα^+^ IELs. Total thymocytes were stained with anti-CD8α, CD25, and CD4 antibodies together with CD1d-tetramer. Live cells were analyzed by excluding doublets. IEL, intraepithelial lymphocyte.
(TIFF)

S4 Fig Frequency of NKT and MAIT cells was not reduced in Rbpj^−/−^ mice. The frequency of (A) NKT cells in the spleen and (B) MAIT cells in inguinal lymph nodes from Rbpj^+/+^ and Rbpj^−/−^ mice as evaluated by flow cytometry. CD1d-tetramer^+^TCRβ^+^ and B220^−^I-A/I-E^−^TCRβ^+^ 5-OP-RU-loaded tetramer-positive cells were defined as NKT and MAIT cells, respectively. The data in (A) and (B) are representative of three independent experiments and are shown as mean ± S.D. Data associated with this figure can be found in the supplemental data file (S1 Data). MAIT, mucosal-associated invariant T; NKT, natural killer T; N.S., not significant; Rbpj, recombination signal binding protein for immunoglobulin kappa J region.
(TIF)

S5 Fig Increased frequency of annexin V–positive cells among TCRαβ^+^CD8αα^+^ IELs isolated from Rbpj^−/−^ mice. Annexin V^+^ cells in TCRαβ^+^CD8αα^+^ IELs, TCRαβ^+^CD8αβ^+^ IELs, and TCRαβ^+^CD4^+^ IELs from Rbpj^+/+^ and Rbpj^−/−^ mice were analyzed by flow cytometry. The data in this figure are representative of three independent experiments and are mean ± S.D., and * indicates *p* < 0.05. Data associated with this figure can be found in the supplemental data file (S1 Data). IEL, intraepithelial lymphocyte; Rbpj, recombination signal binding protein for immunoglobulin kappa J region.
(TIFF)

S6 Fig Cell death and proliferation of TCRαβ^+^CD8αα^+^ IELs. (A) IELs from Rbpj^+/+^ (CD45.1/45.2) and Rbpj^−/−^ (CD45.2) mice were incubated in the presence of IL-7 and IL-15 at a 1:1 ratio of TCRβ^+^ IELs for 4 or 6 d. Then, the ratio of CD45.1- and CD45.2-positive TCRβ^+^CD8α^+^CD4^−^TCRγδ^−^CD8β^−^ IELs was analyzed. Expression of (B) Bcl-2 or (C) Ki-67 in freshly isolated TCRαβ^+^CD8αα^+^ IELs. Shadow: isotype control; solid line: Rbpj^+/+^; dotted line: Rbpj^−/−^. Bcl-2, B cell lymphoma 2; IEL, intraepithelial lymphocyte; IL, interleukin; Rbpj, recombination signal binding protein for immunoglobulin kappa J region.
(TIFF)

S7 Fig Erk and p-Erk expression was not altered in Rbpj^−/−^ mice. The expression of Erk and p-Erk was compared by flow cytometry for TCRαβ^+^CD8αα^+^ IELs isolated from Rbpj^+/+^ and Rbpj^−/−^ mice. Shadow: isotype control; solid line: Rbpj^+/+^; dotted line: Rbpj^+/+^. The data in this figure are representative of three independent experiments. Erk, extracellular signal–regulated kinase; IEL, intraepithelial lymphocyte; p-Erk, phosphorylated Erk; Rbpj, recombination signal binding protein for immunoglobulin kappa J region.
(TIFF)

S1 Methods A description of methods performed to achieve IELs culture and colitis induction. IEL, intraepithelial lymphocyte.
(DOCX)

S1 Data Data underlying Figs 1–6, S2 Fig, S4 Fig and S5 Fig. (XLSX)

## Abbreviations

Ab: antibody
Bcl-2: B cell lymphoma 2
CSF1R: colony stimulating factor 1 receptor
CX_3_CR1: CX_3_C chemokine receptor 1
DC: dendritic cell
Dll1: Delta-like 1
Dll4: Delta-like 4
DSS: dextran sodium sulfate
ERK: extracellular signal–regulated kinase
GFP: green fluorescent protein
IEL: intestinal intraepithelial lymphocyte
IL: interleukin
MAIT: mucosal-associated invariant T
NBD: 7-nitrobenz-2-oxa-1,3-diazol-4-yl
NKT: natural killer T
p-ERK: phosphorylated ERK
PC: phosphatidylcholine
PD-1: programmed death-1
PE: phosphatidylethanolamine
PS: phosphatidylserine
Rbpj: recombination signal binding protein for immunoglobulin kappa J region
SCID: severe combined immunodeficiency
TAK1: TGF-β-activated kinase 1
TCR: T-cell receptor
TGF-β: transforming growth factor β

## References

[1] Izcue A, Coombes JL, Powrie F. Regulatory lymphocytes and intestinal inflammation. Annu Rev Immunol. 2009;27:313–38. 10.1146/annurev.immunol.021908.132657 . 19302043

[2] Mowat AM, Agace WW. Regional specialization within the intestinal immune system. Nat Rev Immunol. 2014;14(10):667–85. 10.1038/nri3738 . 25234148

[3] Caballero S, Pamer EG. Microbiota-mediated inflammation and antimicrobial defense in the intestine. Annu Rev Immunol. 2015;33:227–56. 10.1146/annurev-immunol-032713-120238 25581310 PMC4540477

[4] Cheroutre H, Lambolez F, Mucida D. The light and dark sides of intestinal intraepithelial lymphocytes. Nat Rev Immunol. 2011;11(7):445–56. 10.1038/nri3007 21681197 PMC3140792

[5] Poussier P, Ning T, Banerjee D, Julius M. A unique subset of self-specific intraintestinal T cells maintains gut integrity. J Exp Med. 2002;195(11):1491–7. Epub 2002/06/05. PubMed Central PMCID: PMC2193537. 10.1084/jem.20011793 12045247 PMC2193537

[6] van Wijk F, Cheroutre H. Intestinal T cells: facing the mucosal immune dilemma with synergy and diversity. Semin Immunol. 2009;21(3):130–8. 10.1016/j.smim.2009.03.003 19386513 PMC2794834

[7] Yamagata T, Mathis D, Benoist C. Self-reactivity in thymic double-positive cells commits cells to a CD8 alpha alpha lineage with characteristics of innate immune cells. Nat Immunol. 2004;5(6):597–605. 10.1038/ni1070 . 15133507

[8] Leishman AJ, Gapin L, Capone M, Palmer E, MacDonald HR, Kronenberg M, et al Precursors of functional MHC class I- or class II-restricted CD8alphaalpha(+) T cells are positively selected in the thymus by agonist self-peptides. Immunity. 2002;16(3):355–64. . 11911821 10.1016/s1074-7613(02)00284-4

[9] Ruscher R, Kummer RL, Lee YJ, Jameson SC, Hogquist KA. CD8alphaalpha intraepithelial lymphocytes arise from two main thymic precursors. Nat Immunol. 2017;18(7):771–9. 10.1038/ni.3751 28530714 PMC5505317

[10] Jiang W, Ferrero I, Laurenti E, Trumpp A, MacDonald HR. c-Myc controls the development of CD8alphaalpha TCRalphabeta intestinal intraepithelial lymphocytes from thymic precursors by regulating IL-15-dependent survival. Blood. 2010;115(22):4431–8. Epub 2010/03/24. 10.1182/blood-2009-11-254698 . 20308599

[11] Konkel JE, Maruyama T, Carpenter AC, Xiong Y, Zamarron BF, Hall BE, et al Control of the development of CD8alphaalpha+ intestinal intraepithelial lymphocytes by TGF-beta. Nat Immunol. 2011;12(4):312–9. 10.1038/ni.1997 21297643 PMC3062738

[12] Ma LJ, Acero LF, Zal T, Schluns KS. Trans-presentation of IL-15 by intestinal epithelial cells drives development of CD8alphaalpha IELs. J Immunol. 2009;183(2):1044–54. Epub 2009/06/26. 10.4049/jimmunol.0900420 19553528 PMC2706935

[13] Radtke F, Fasnacht N, Macdonald HR. Notch signaling in the immune system. Immunity. 2010;32(1):14–27. 10.1016/j.immuni.2010.01.004 . 20152168

[14] Radtke F, MacDonald HR, Tacchini-Cottier F. Regulation of innate and adaptive immunity by Notch. Nat Rev Immunol. 2013;13(6):427–37. 10.1038/nri3445 . 23665520

[15] Maekawa Y, Minato Y, Ishifune C, Kurihara T, Kitamura A, Kojima H, et al Notch2 integrates signaling by the transcription factors RBP-J and CREB1 to promote T cell cytotoxicity. Nat Immunol. 2008;9(10):1140–7. Epub 2008/08/30. 10.1038/ni.1649 . 18724371

[16] Maekawa Y, Ishifune C, Tsukumo S, Hozumi K, Yagita H, Yasutomo K. Notch controls the survival of memory CD4+ T cells by regulating glucose uptake. Nat Med. 2015;21(1):55–61. 10.1038/nm.3758 . 25501905

[17] Ishifune C, Maruyama S, Sasaki Y, Yagita H, Hozumi K, Tomita T, et al Differentiation of CD11c+ CX3CR1+ cells in the small intestine requires Notch signaling. Proc Natl Acad Sci U S A. 2014;111(16):5986–91. 10.1073/pnas.1401671111 24711412 PMC4000843

[18] Nakajima K, Maekawa Y, Kataoka K, Ishifune C, Nishida J, Arimochi H, et al The ARNT-STAT3 axis regulates the differentiation of intestinal intraepithelial TCRalphabeta(+)CD8alphaalpha(+) cells. Nat Commun. 2013;4:2112 Epub 2013/07/10. 10.1038/ncomms3112 . 23836150

[19] Stamataki D, Holder M, Hodgetts C, Jeffery R, Nye E, Spencer-Dene B, et al Delta1 expression, cell cycle exit, and commitment to a specific secretory fate coincide within a few hours in the mouse intestinal stem cell system. PLoS ONE. 2011;6(9):e24484 10.1371/journal.pone.0024484 21915337 PMC3168508

[20] Sekine C, Moriyama Y, Koyanagi A, Koyama N, Ogata H, Okumura K, et al Differential regulation of splenic CD8- dendritic cells and marginal zone B cells by Notch ligands. Int Immunol. 2009;21(3):295–301. 10.1093/intimm/dxn148 . 19181931

[21] Lai YG, Hou MS, Hsu YW, Chang CL, Liou YH, Tsai MH, et al IL-15 does not affect IEL development in the thymus but regulates homeostasis of putative precursors and mature CD8 alpha alpha+ IELs in the intestine. J Immunol. 2008;180(6):3757–65. Epub 2008/03/07. . 18322181 10.4049/jimmunol.180.6.3757

[22] Wang HC, Zhou Q, Dragoo J, Klein JR. Most murine CD8+ intestinal intraepithelial lymphocytes are partially but not fully activated T cells. J Immunol. 2002;169(9):4717–22. . 12391179 10.4049/jimmunol.169.9.4717

[23] El-Asady R, Yuan R, Liu K, Wang D, Gress RE, Lucas PJ, et al TGF-{beta}-dependent CD103 expression by CD8(+) T cells promotes selective destruction of the host intestinal epithelium during graft-versus-host disease. J Exp Med. 2005;201(10):1647–57. 10.1084/jem.20041044 15897278 PMC2212926

[24] Guy-Grand D, Vassalli P. Gut intraepithelial T lymphocytes. Curr Opin Immunol. 1993;5(2):247–52. . 8507401 10.1016/0952-7915(93)90012-h

[25] Segawa K, Kurata S, Nagata S. Human Type IV P-type ATPases That Work as Plasma Membrane Phospholipid Flippases and Their Regulation by Caspase and Calcium. J Biol Chem. 2016;291(2):762–72. 10.1074/jbc.M115.690727 26567335 PMC4705396

[26] Toda S, Nishi C, Yanagihashi Y, Segawa K, Nagata S. Clearance of Apoptotic Cells and Pyrenocytes. Curr Top Dev Biol. 2015;114:267–95. 10.1016/bs.ctdb.2015.07.017 . 26431571

[27] Xu C, Gagnon E, Call ME, Schnell JR, Schwieters CD, Carman CV, et al Regulation of T cell receptor activation by dynamic membrane binding of the CD3epsilon cytoplasmic tyrosine-based motif. Cell. 2008;135(4):702–13. 10.1016/j.cell.2008.09.044 19013279 PMC2597348

[28] Gagnon E, Schubert DA, Gordo S, Chu HH, Wucherpfennig KW. Local changes in lipid environment of TCR microclusters regulate membrane binding by the CD3epsilon cytoplasmic domain. J Exp Med. 2012;209(13):2423–39. 10.1084/jem.20120790 23166358 PMC3526357

[29] Shi X, Bi Y, Yang W, Guo X, Jiang Y, Wan C, et al Ca2+ regulates T-cell receptor activation by modulating the charge property of lipids. Nature. 2013;493(7430):111–5. 10.1038/nature11699 . 23201688

[30] Zhu X, Libby RT, de Vries WN, Smith RS, Wright DL, Bronson RT, et al Mutations in a P-type ATPase gene cause axonal degeneration. PLoS Genet. 2012;8(8):e1002853 10.1371/journal.pgen.1002853 22912588 PMC3415440

[31] Lee CS, Penberthy KK, Wheeler KM, Juncadella IJ, Vandenabeele P, Lysiak JJ, et al Boosting Apoptotic Cell Clearance by Colonic Epithelial Cells Attenuates Inflammation In Vivo. Immunity. 2016;44(4):807–20. 10.1016/j.immuni.2016.02.005 27037190 PMC4838559

[32] Asano K, Takahashi N, Ushiki M, Monya M, Aihara F, Kuboki E, et al Intestinal CD169(+) macrophages initiate mucosal inflammation by secreting CCL8 that recruits inflammatory monocytes. Nat Commun. 2015;6:7802 10.1038/ncomms8802 26193821 PMC4518321

[33] Niess JH, Brand S, Gu X, Landsman L, Jung S, McCormick BA, et al CX3CR1-mediated dendritic cell access to the intestinal lumen and bacterial clearance. Science. 2005;307(5707):254–8. 10.1126/science.1102901 . 15653504

[34] Wang X, Sumida H, Cyster JG. GPR18 is required for a normal CD8alphaalpha intestinal intraepithelial lymphocyte compartment. J Exp Med. 2014;211(12):2351–9. 10.1084/jem.20140646 25348153 PMC4235638

[35] Amsen D, Blander JM, Lee GR, Tanigaki K, Honjo T, Flavell RA. Instruction of distinct CD4 T helper cell fates by different notch ligands on antigen-presenting cells. Cell. 2004;117(4):515–26. . 15137944 10.1016/s0092-8674(04)00451-9

[36] Alam MS, Maekawa Y, Kitamura A, Tanigaki K, Yoshimoto T, Kishihara K, et al Notch signaling drives IL-22 secretion in CD4+ T cells by stimulating the aryl hydrocarbon receptor. Proc Natl Acad Sci U S A. 2010;107(13):5943–8. Epub 2010/03/17. 10.1073/pnas.0911755107 20231432 PMC2851859

[37] Backer RA, Helbig C, Gentek R, Kent A, Laidlaw BJ, Dominguez CX, et al A central role for Notch in effector CD8(+) T cell differentiation. Nat Immunol. 2014;15(12):1143–51. 10.1038/ni.3027 25344724 PMC4232996

[38] Han H, Tanigaki K, Yamamoto N, Kuroda K, Yoshimoto M, Nakahata T, et al Inducible gene knockout of transcription factor recombination signal binding protein-J reveals its essential role in T versus B lineage decision. Int Immunol. 2002;14(6):637–45. . 12039915 10.1093/intimm/dxf030

[39] Saito T, Chiba S, Ichikawa M, Kunisato A, Asai T, Shimizu K, et al Notch2 is preferentially expressed in mature B cells and indispensable for marginal zone B lineage development. Immunity. 2003;18(5):675–85. . 12753744 10.1016/s1074-7613(03)00111-0

[40] Estrach S, Ambler CA, Lo Celso C, Hozumi K, Watt FM. Jagged 1 is a beta-catenin target gene required for ectopic hair follicle formation in adult epidermis. Development. 2006;133(22):4427–38. 10.1242/dev.02644 . 17035290

[41] Hozumi K, Negishi N, Suzuki D, Abe N, Sotomaru Y, Tamaoki N, et al Delta-like 1 is necessary for the generation of marginal zone B cells but not T cells in vivo. Nat Immunol. 2004;5(6):638–44. 10.1038/ni1075 . 15146182

[42] Reantragoon R, Corbett AJ, Sakala IG, Gherardin NA, Furness JB, Chen Z, et al Antigen-loaded MR1 tetramers define T cell receptor heterogeneity in mucosal-associated invariant T cells. J Exp Med. 2013;210(11):2305–20. 10.1084/jem.20130958 24101382 PMC3804952

[43] Dolton G, Tungatt K, Lloyd A, Bianchi V, Theaker SM, Trimby A, et al More tricks with tetramers: a practical guide to staining T cells with peptide-MHC multimers. Immunology. 2015;146(1):11–22. 10.1111/imm.12499 26076649 PMC4552497

