# Supplementary material for: Regulation of membrane phospholipid asymmetry by Notch-mediated flippase expression controls the number of intraepithelial TCRαβ+CD8αα+ T cells
Source: PLoS Biol. 2019 May 9;17(5):e3000262. doi: 10.1371/journal.pbio.3000262 (PMC6529014; doi:10.1371/journal.pbio.3000262)
Supplement: S1 Methods — IEL, intraepithelial lymphocyte. (DOCX) [file pbio.3000262.s008.docx]

**Supplementary methods**

**IELs culture**

An equal ratio of TCRβ^+^ IELs from Rbpj^+/+^ (CD45.1/45.2) and Rbpj^-/-^ (CD45.2) mice was incubated in in the presence of IL-7 (10 ng/ml, R&D Systems) and IL-15 (100 ng/ml, R&D Systems). The ratio of CD45.1- and CD45.2-positive TCRαβ^+^CD8αα^+^ IELs was analyzed 4 or 6 days after culture.

**Colitis**

For DSS-induced colitis, drinking water with 2% DSS (molecular weight: 36,000–50,000 Da; MP Biomedicals) was administered on day 0-day 7. On days 2 and 4, 2% DSS was replenished with fresh stocks. For TNBS-induced colitis, the backs of mice were painted with 150 μl of 1% (w/v) TNBS diluted in acetone:olive oil (4:1) solution. Seven days later, mice that had been fasted overnight were treated with 100 μl of 5% (w/v) TNBS injected into the anus.
